# Behavioral and biologic characteristics of cancer-related cognitive impairment biotypes

**DOI:** 10.1007/s11682-023-00774-6

**Published:** 2023-05-02

**Authors:** Michele M. Mulholland, Sarah Prinsloo, Elizabeth Kvale, Adrienne N. Dula, Oxana Palesh, Shelli R. Kesler

**Affiliations:** 1 Keeling Center for Comparative Medicine and Research, The University of Texas MD Anderson Cancer Center, Bastrop, TX USA; 2 Department of Neurosurgery, The University of Texas MD Anderson Cancer Center, Houston, TX USA; 3 Department of Geriatrics and Palliative Care, Baylor College of Medicine, Houston, TX USA; 4 Department of Neurology, Dell School of Medicine, The University of Texas at Austin, Austin, TX USA; 5 Department of Psychiatry, Massey Cancer Center, Virginia Commonwealth University School of Medicine, Richmond,, VA USA; 6 Department of Adult Health, School of Nursing, The University of Texas at Austin, 1710 Red River St, D0100, Austin, TX 78712 USA

**Keywords:** Breast cancer, Chemotherapy, Chemotherapy-related cognitive impairment, Biotypes, Neurofunctional stability

## Abstract

Psychiatric diagnosis is moving away from symptom-based classification and towards multi-dimensional, biologically-based characterization, or biotyping. We previously identified three biotypes of chemotherapy-related cognitive impairment based on functional brain connectivity. In this follow-up study of 80 chemotherapy-treated breast cancer survivors and 80 non-cancer controls, we evaluated additional factors to help explain biotype expression: neurofunctional stability, brain age, apolipoprotein (APOE) genotype, and psychoneurologic symptoms. We also compared the discriminative ability of a traditional, symptom-based cognitive impairment definition with that of biotypes. We found significant differences in cortical brain age (F = 10.50, p < 0.001), neurofunctional stability (F = 2.83, p = 0.041), APOE e4 genotype (X^2^ = 7.68, p = 0.050), and psychoneurological symptoms (Pillai = 0.378, p < 0.001) across the three biotypes. The more resilient Biotype 2 demonstrated significantly higher neurofunctional stability compared to the other biotypes. Symptom-based classification of cognitive impairment did not differentiate biologic or other behavioral variables, suggesting that traditional categorization of cancer-related cognitive effects may miss important characteristics which could inform targeted treatment strategies. Additionally, biotyping, but not symptom-typing, was able to distinguish survivors with cognitive versus psychological effects. Our results suggest that Biotype 1 survivors might benefit from first addressing symptoms of anxiety and fatigue, Biotype 3 might benefit from a treatment plan which includes sleep hygiene, and Biotype 2 might benefit most from cognitive skills training or rehabilitation. Future research should include additional demographic and clinical information to further investigate biotype expression related to risk and resilience and examine integration of more clinically feasible imaging approaches.

## Introduction

Patients with cancer frequently experience persistent cognitive impairment following cancer therapies (Mayo et al. 2021). The etiology of cancer-related cognitive impairment (CRCI) remains poorly understood, partly due to the lack of a clear definition of this syndrome. Clinical management of CRCI in terms of prognosis, prevention, and treatment is also hindered without a definition of CRCI. Traditional assessment of CRCI relies on symptom information obtained from neuropsychological testing and patient self-report. However, there is no standard threshold for impaired performance.

Knowledge regarding the mechanisms underlying symptoms is necessary for precision medicine but symptom assessments do not provide this information (Miranda et al. 2021). Psychiatric diagnosis is therefore moving away from symptom-based classification and toward biologically driven definitions. Examples of these initiatives include the National Institutes of Health’s Research Domain Criteria (RDoC) (Insel et al. 2010) and the Bipolar-Schizophrenia Network for Intermediate Phenotypes (B-SNIP) (Tamminga et al. 2013). These initiatives aim to determine the neurobiologic signatures of distinct symptom clusters to provide precise, dimensional classification of psychiatric syndromes. For example, B-SNIP researchers have identified three distinct, neurophysiologic subtypes, or biotypes, of psychosis (Clementz et al. 2016). Other groups have identified biotypes of depression (Drysdale et al. 2017), Parkinson’s disease (Wang et al. 2020), and multiple sclerosis (Pontillo et al. 2022).

We previously identified three biotypes of CRCI using measures of functional brain connectivity in a sample of 80 chemotherapy-treated breast cancer survivors (Kesler et al. 2020). Biotype 1 demonstrated low cognitive function, Biotype 2 had relatively preserved cognitive function, and Biotype 3 showed moderately low cognitive function. Biotype 1 consisted of significantly more racial and ethnic minority survivors suggesting increased vulnerability to cognitive effects related to potential health disparities. Surprisingly, Biotype 2 was characterized by significantly higher disease severity and treatment intensity. Biotype 3 was characterized by significantly longer time off treatment compared to Biotype 1, which may explain the improved cognitive functioning compared to Biotype 1. However, we could find no other features that explained biotype expression including age, menopausal status, or education level.

The aim of this follow-up study was to evaluate additional factors that may help explain biotype expression. We measured new biological characteristics including neurofunctional stability, brain age and apolipoprotein (APOE) genotype. The flexibility of functional brain networks is necessary for learning and adaptation but must be constrained to provide the stability required for retaining information and minimizing the load on physiological resources (Hermundstad et al. 2011; Bassett et al. 2011). Stability of functional networks can be measured by examining the fractal quality of their underlying functional signal (Ciuciu et al. 2014; Ciuciu et al. 2012). Our group and others have observed disrupted neurofunctional stability in patients with breast cancer (Kesler et al. 2017; Churchill et al. 2015).

Aging, neurodegeneration, and cancer share many underlying mechanisms and therefore, altered, or accelerated aging may play a role in cancer-related cognitive impairment (Mandelblatt et al. 2013; Chang et al. 2019). Machine learning algorithms can be used to estimate brain age from non-invasive neuroimaging acquisitions (Liem et al. 2017). By employing this technique, we previously demonstrated increased cortical aging in patients with breast cancer from pre- to post-chemotherapy treatment (Henneghan et al. 2020).

The APOE e4 genotype is associated with impaired neuronal membrane repair and synaptic plasticity, increased amyloid-β deposition, tau pathology, neuroinflammation, and disrupted cerebrovascular function (White et al. 2001; Shi et al. 2017; Zlokovic 2013). Accordingly, carriers of this allele are at increased risk for neurodegenerative conditions that affect cognition. Our group and others have shown that the APOE e4 genotypic variant is associated with lower cognitive function in breast cancer (Harrison et al. 2021; Speidell et al. 2019; Ahles et al. 2014). We also previously demonstrated a potentially altered relationship between APOE genotype and neurogenesis in patients with breast cancer (Harrison et al. 2021) and there is some evidence suggesting an association between APOE e4 genotype and breast cancer pathogenesis (Cibeira et al. 2014).

Other psychoneurologic symptoms (anxiety, depression, disrupted sleep) tend to co-occur with cognitive impairment and it can be difficult to disentangle these (Mandelblatt et al. 2020). Determining characteristics associated with vulnerability and resilience may point to modifiable factors that could help reduce symptoms of CRCI. We hypothesized that the resilient Biotype 2 survivors would demonstrate greater functional brain network stability, lower cortical brain age, lower frequency of APOE e4 genotype, and fewer psychoneurologic symptoms compared to the other biotypes. We also compared the discriminative ability of traditional symptom-based cognitive impairment definition with that of biotypes.

## Methods

### Participants

We utilized our prior sample of 80 chemotherapy-treated, primary breast cancer survivors (Kesler et al. 2020). These women were age 35–73 years and had completed all primary treatments (surgery, radiation, chemotherapy) excluding hormone blockade at least 6 months before study enrollment (Table 1). Chemotherapy regimens included doxorubicin/cyclophosphamide (N = 3), doxorubicin/cyclophosphamide/paclitaxel (N = 52), doxorubicin/paclitaxel (N = 1), doxorubicin/cyclophosphamide/fluorouracil (N = 1), doxorubicin/cyclophosphamide/methotrexate (N = 5), cyclophosphamide/paclitaxel (N = 16), and fluorouracil/epirubicin/cyclophosphamide (N = 2). Participants were free from disease and had no history of relapse or recurrence at the time of evaluation. Participants were excluded for neurologic, psychiatric, or medical conditions known to affect cognitive function. As noted above, participants were previously clustered into 3 biotypes (Biotype 1: “Low cognitive function”,, Biotype 2: “Cognitively resilient”, Biotype 3: “Moderately low cognitive function”) based on their individual patterns of functional connectivity within 8 functional brain networks, Biotype 1 (N = 36), Biotype 2 (N = 24) and Biotype 3 (N = 20) (Kesler et al. 2020). Also included in the prior study were 82 non-cancer controls which we used again for comparison in the present study (Table 1). Two controls met criteria for cognitive impairment so were excluded from this study. This study was approved by the Stanford University Institutional Review Board and all participants provided written informed consent.

**Table 1:** Biotype Characteristics. Data are shown as mean (standard deviation) unless otherwise noted

	Biotype 1 (N = 36)	Biotype 2 (N = 24)	Biotype 3 (N = 20)	Controls (N = 80)	Stat	P value
Age (years)	49.30 (8.0)	52.52 (6.4)	52.16 (8.7)	49.29 (13.2)	F = 0.802	0.494
Education (years)	16.39 (2.4)	17.13 (2.7)	16.47 (2.2)	16.99 (2.4)	F = 0.782	0.506
Racial/ethnic minority (%)	36%	13%	12%	16%	X^2^ = 8.70	0.033
Post-menopause (%)	61%	71%	59%	67%	X^2^ = 1.09	0.779
Stage at diagnosis (I,II,III %)	30%,65%,5%	21%,41%,38%	18%,65%,17%		X^2^ = 5.42	0.247
Radiotherapy (%)	67%	96%	65%		X^2^ = 7.91	0.019
Hormone blockade (%)	61%	75%	64%		X^2^ = 1.27	0.531
Months since chemotherapy ended	26.40 (18.7)	49.67 (33.9)	64.25 (82.1)		F = 4.69	0.012

#### Neuroimaging data acquisitions

Functional magnetic resonance imaging (fMRI) data were obtained while participants rested with eyes closed using a T2* weighted gradient echo spiral pulse sequence: TR = 2000 ms, TE = 30 ms, flip angle = 80°, and 1 interleave, FOV = 22 cm, matrix = 64 × 64, in-plane resolution = 3.4375 mm^2^, number of volumes = 216 with a 3 T GE Signa HDx whole body scanner (GE Medical Systems, Milwaukee, WI). A high-order shimming method was employed to reduce field heterogeneity. A high-resolution, 3D IR-prepared FSPGR anatomic scan was obtained: TR: 8.5, TE: minimum, flip: 15 degrees, TI: 400 ms, BW: + / − 31.25 kHz, FOV: 22 cm, phase FOV: 0.75, slice thickness: 1.5 mm, 124 slices, 256 × 256 @ 1 NEX, scan time: 4:33 min.

#### Neurofunctional stability

Spontaneous functional time series were measured from resting state fMRI data using Statistical Parametric Mapping 12 (Friston et al. 1994) and CONN 21a (Whitfield-Gabrieli and Nieto-Castanon 2012) implemented in Matlab v2021b (Mathworks, Inc, Natick, MA). Briefly, this involved realignment, coregistration with the segmented anatomic volume, spatial normalization, artifact detection (global signal = 3.0 standard deviations, motion = 1.0 mm, rotation = 0.05 mm), band-pass filtering (0.008–0.09 Hz), and correction of non-neuronal noise (Behzadi et al. 2007). We evaluated the temporal stability of spontaneous brain function by calculating the mean rescaled range Hurst exponent (Hurst 1951) across the entire brain. The windowing function was based on a data-derived natural number that possessed the largest number of divisors among all natural numbers in the time series interval. The Hurst exponent quantifies how correlated a time series is with itself, or how well it reflects elements of the baseline signal from both the recent and remote past. Although biotypes were originally defined based on functional connectivity, the Hurst exponent is based on dynamics of the time series and not the static correlation between regions. Hurst was not correlated with functional connectivity properties in breast cancer or controls (Kesler et al. 2017), nor with the connectivity features used for biotyping (r < 0.097, p > 0.26).

#### Brain age

Cortical thickness was measured from the anatomic volume using FreeSurfer (Fischl 2012). Briefly, non-brain tissue was removed followed by an automated spatial transformation, segmentation, intensity normalization, tessellation of gray/white-matter boundary, automated correction of topological defects and surface deformation to form the gray and white matter boundary. Cortical thickness was determined as the difference between the pial and white-matter surface (Fischl et al. 2002; Dale et al. 1999). We performed visual quality checks to ensure no major errors within the automated processing. Cortical age was then estimated using the Brain-Age Regression Analysis and Computation Utility Software (Liem et al. 2017).

#### APOE genotype

Saliva samples were obtained from all participants using the Oragene DNA OG-250 collection kit (DNA Genotek, Kanata, Ontario). Genotyping was accomplished by polymerase chain reaction (PCR) fragment length polymorphism analysis with restricted fragment length polymorphisms. Twenty-three breast cancer survivors and 30 controls opted out of providing a saliva sample.

#### Psychoneurological symptoms

Depression, anxiety, and fatigue were measured using the Clinical Assessment of Depression (CAD) (Aghakhani et al. 2004). We previously evaluated the total score for this questionnaire and found no biotype differences (Kesler et al. 2020), but here we examined subscales for a more granular assessment of symptoms. We measured sleep disruption using the Pittsburgh Sleep Quality Index (PSQI) (Buysse et al. 1989).

#### Symptom-based categories

Our previous study examined cognitive symptoms between biotypes using a battery of five standardized cognitive tests (Kesler et al. 2020). For the present study, we employed a common symptom-based classification to determine impairment categories (Weffel et al. 2011). Specifically, a participant was classified as impaired if 2 or more cognitive test scores were 1.5 or more standard deviations below the test’s normative mean or at least one test score was 2 or more standard deviations below the normative mean. This classification will be referred to hereafter as *symptom-type*; 26% percent of participants were classified as having an impaired symptom-type and the remaining 74% had a non-impaired symptom-type (Table 2).

**Table 2:** Symptom-Type Characteristics. Data are shown as mean (standard deviation) unless otherwise noted

	Unimpaired (N = 59)	Impaired (N = 21)	Controls (N = 80)	Stat	P value
Age (years)	51.72 (7.1)	48.59 (9.0)	49.29 (13.2)	F = 1.08	0.343
Education (years)	16.88 (2.5)	15.95 (2.5)	16.99 (2.4)	F = 1.55	0.215
Racial/ethnic minority (%)	12%	55%	16%	X^2^ = 19.1	< 0.001
Post-menopause (%)	65%	60%	67%	X^2^ 0.421	0.810
Stage at diagnosis (I,II,III %)	21%,56%,23%	25%,55%,20%		X^2^ = 0.161	0.923
Radiotherapy (%)	75%	75%		X^2^ = 0.002	0.969
Hormone blockade (%)	70%	55%		X^2^ = 1.52	0.217
Months since chemotherapy ended	48.5 (52.6)	23.4 (7.5)		F = 4.50	0.037

#### Statistical analyses

Data were first inspected visually to confirm normality and homogeneity of variance. Brain network stability, cortical brain age, and psychoneurological symptoms were compared between the biotypes or symptom-types using ANOVA with Tukey correction for post-hoc pairwise biotype comparisons. APOE genotype was compared between the biotypes or symptom-types using a chi-squared test. CAD subscales were evaluated between biotypes or symptom-types using MANOVA followed by ANOVA with Tukey correction. PSQI scores were compared using ANOVA with Tukey correction.

## Results

### Neurofunctional stability

**Fig. 1:**
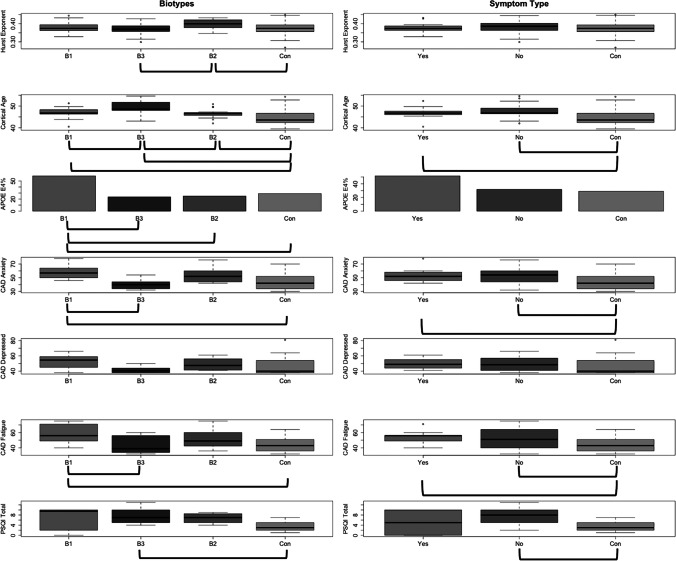
Biological and Behavioral Characteristics by Biotype and Symptom-Type. Left Column: Neurofunctional stability, brain age and apolipoprotein (APOE) e4 genotype differed significantly among biotypes and between biotypes and controls. Biotype 2 showed higher neurofunctional stability compared to both Biotype 3 and non-cancer controls. Brain age was significantly higher for all three biotypes compared to controls; additionally, Biotype 3 had significantly higher brain age compared to both Biotypes 1 and 2. Biotype 1 patients had a higher percentage of APOE E4 genotype compared to Biotypes 2, 3, and non-cancer controls. Biotype 1 had significantly higher anxiety levels and fatigue compared to both Biotype 3 and controls. However, only Biotype 3 had significantly more sleep disruption compared to controls. Right column: Brain age differed between both symptom-types (impaired/not impaired) and controls but not between the two symptom-types. Both symptom-types (impaired/not impaired) had significantly higher anxiety and fatigue compared to controls; however, these did not differ between symptom-types. Only those with unimpaired symptom type had significantly higher sleep disruption compared to controls. Brackets indicate significant difference (p < 0.05). B1: Biotype 1: “Low cognitive function”, B2: Biotype 2: “Cognitively resilient”, B3: Biotype 3: “Moderately low cognitive function”, Con: controls, Yes: impaired symptom-type, No: unimpaired symptom-type. CAD: Clinical Assessment of Depression; PSQI: Pittsburg Sleep Quality Index; APOE E4: apolipoprotein e4 allele

Biotype 2 showed significantly elevated Hurst exponent compared to Biotype 3 and to controls (F = 2.83, p = 0.041, Fig. 1). Hurst exponent did not differ between symptom-types (F = 1.024, p = 0.362, Fig. 1).

### Brain age

Cortical age was significantly higher in Biotype 3 compared to Biotypes 1 and 2 and was higher in all biotypes compared to controls (F = 10.50, p < 0.001, Fig. 1). Cortical age did not differ between symptom-types, but was significantly different between symptom-types and controls (F = 10.74, p < 0.001, Fig. 1).

### APOE genotype

The incidence of the APOE e4 genotype was significantly higher in Biotype 1 compared to the other biotypes and controls (X^2^ = 7.68, p = 0.050, Fig. 1). Genotype did not quite differ by symptom-type (F = 4.50, p = 0.106), although the impaired symptom-type was associated with higher frequency of the APOE e4 allele (Fig. 1).

### Psychoneurological symptoms

The MANOVA comparing CAD subscales among biotypes and controls was significant (Pillai = 0.378, p < 0.001). Biotype 1 demonstrated significantly higher anxiety (F = 7.31, p < 0.001) and fatigue (F = 6.89, p < 0.001) compared to Biotype 3 and controls (Fig. 1). Depression did not differ significantly between the biotypes or controls (F = 2.62, p = 0.069). Sleep disruption was significantly higher in Biotype 3 compared to controls (F = 3.34, p = 0.025, Fig. 1).

The MANOVA comparing CAD subscales among symptom-types and controls was significant (Pillai = 0.250, p = 0.028). Anxiety did not differ between symptom-types but was higher in both symptom-types compared to controls (F = 5.03, p = 0.009). Similarly, fatigue was higher in both symptom-types compared to controls (F = 5.41, p = 0.007) but did not differ between symptom-types. Depression did not differ between symptom-types or controls (F = 1.07, p = 0.348). The non-impaired symptom-type showed significantly higher sleep disruption compared to controls (F = 5.03, p = 0.009, Fig. 1).

## Discussion

Overall, we found significant differences in cortical brain age, functional brain network stability, APOE e4 genotype, and psychoneurological symptoms across the three previously identified (based on functional connectivity) biotypes of CRCI. In contrast, these biological characteristics did not differ between the cognitive symptom-types (impaired/not impaired), however, the cognitive symptom-types did differ from controls in cortical brain age and psychoneurological symptoms (specifically anxiety and fatigue). These findings indicate that classical categorization of cognitively impaired or non-impaired breast cancer survivors may miss important biological characteristics which could potentially be used to 1) predict prognosis, and 2) inform more targeted treatment strategies.

Consistent with our hypothesis, Biotype 2 showed greater functional stability than the other biotypes, but this was significantly higher than controls, suggesting it may be overly stable (i.e., inflexible) in some respects. We previously showed that functional stability was lower in patients with breast cancer prior to initiation of anti-cancer treatment (Kesler et al. 2017). There is a trade-off between functional stability and flexibility, with a healthy balance needed for efficient information processing and an imbalance associated with several diseases and disorders (Wei et al. 2013; Maxim et al. 2005; Sokunbi et al. 2014; Liljenström 2003). Elevated neurofunctional stability has been associated with obsessive or inflexible thinking in other populations (Gao et al. 2022). Like all behaviors, obsessive thinking exists on a continuum where it becomes detrimental in the extreme but can be useful in smaller “doses”, such as always putting one’s keys and wallet in the exact same location to compensate for forgetfulness. It is possible that Biotype 2 survivors engage in some degree of obsessive thinking and behavior that improve their cognitive performance. Compensatory strategies such as this are strongly encouraged in individuals with cognitive problems, and our prior work suggests that compensatory strategy usage should be incorporated into the assessment of CRCI (Kesler et al. 2022). However, the neural correlates of their use are currently unknown. The precise manner in which increased neurofunctional stability contributes to preservation of cognitive function in Biotype 2 requires further study.

Biotype 2 did not differ in anxiety, fatigue, sleep disruption, or depression, when compared to the other biotypes or controls. In addition, Biotype 2 (along with Biotype 3 and controls) showed significantly lower incidence of APOE e4 genotype compared to Biotype 1. Although all three biotypes had significantly higher brain age compared to controls, Biotype 2 had significantly lower brain age than Biotype 3 (those with moderately low cognitive functioning), but surprisingly brain age did not differ between Biotype 2 and Biotype 1 (those with the lowest cognitive functioning). Therefore, the potential characteristics that help explain the relative cognitive resilience of Biotype 2 survivors remains unclear beyond elevated neurofunctional stability.

Biotype showed stronger separation of survivor characteristics compared to cognitive symptom-type. While the latter separated between breast cancer survivors and controls in terms of brain age and psychological function, it did not separate these characteristics between cognitive impaired and non-impaired survivors. Additionally, cognitive symptom-type did not separate survivors from controls in terms of neurofunctional stability or genotype. A previous study by Ji et al. (2020) demonstrated similar results; their electroencephalogram (EEG)-based biotypes were superior to Diagnostic and Statistical Manual (DSM-IV) diagnoses in discriminating regional brain homogeneity in patients with psychosis (Ji et al. 2020). Leikauf et al. (2017) identified two cognitive biotypes of Attention-Deficit Hyperactivity Disorder with corresponding EEG and ECG profiles, unlike the DSM-subtypes. There was also some limited evidence that cognitive patterns in response to treatment differed between the biotypes, but not between the DSM-subtypes (Leikauf et al. 2017). Several studies on depression and anxiety reveal similar findings; multi-model characterizations of patients using biotyping are better at characterizing patients compared to symptomology and are likely to be more useful in determining effective treatment strategies (Goldstein-Piekarski et al. 2022; Williams 2016, 2017; Keller et al. 2020).

The impaired symptom-type was composed largely (90%) of Biotype 1 participants suggesting that it successfully captured survivors with the lowest cognitive function, highest genetic risk, and most psychological distress. Cognitive impairment has been difficult to distinguish from psychological distress in patients with cancer, resulting in some controversy regarding the existence of CRCI. In other words, many patients have been told that CRCI is “just stress”. Our results suggest that a subgroup of particularly vulnerable breast cancer survivors demonstrate both cognitive impairment and psychological distress (Biotype 1) while other subgroups demonstrate cognitive impairment in the absence of significant psychological distress (Biotypes 2 and 3). Biotype 1 survivors would likely benefit from first addressing symptoms of anxiety and fatigue to determine if co-occurring cognitive impairments would improve or even resolve. Biotypes 2 and 3 would be more likely to benefit from a treatment plan focused on cognitive skills rather than psychological distress. With a larger sample, symptom-type may show improved discrimination of characteristics.

For symptom-type classification, those classified as cognitively unimpaired had significantly greater sleep deprivation than those classified as impaired. However, biotyping identified a subgroup (Biotype 3) that was characterized by sleep disruption and moderately impaired cognitive function, even though these survivors had the longest time to recover post-treatment (Kesler et al. 2020). Studies of sleep disruption after brain injuries have revealed that lower sleep quality and more fragmented sleep slows recovery and results in poorer treatment outcomes (Lucke-Wold et al. 2015; Fleming et al. 2020; Iddagoda et al. 2020). It is possible that sleep deprivation has slowed the cognitive recovery of Biotype 3 and therefore a treatment plan including sleep hygiene may be indicated for these breast cancer survivors. Biotype 3 also demonstrated the highest brain age. Sleep disruption scores and brain age were not correlated (r = 0.297, p = 0.204) although Biotype 3 was the smallest group at N = 20 and therefore, we likely lacked power to determine this relationship. Even in healthy adults, sleep deprivation is related to greater age-related brain changes (André et al. 2021; Lo et al. 2014; Krause et al. 2017). It is important to note that it is unclear whether sleep deprivation causes or is a result of increased brain aging. Prospective, longitudinal studies are needed to further illuminate this relationship, and how it may impact breast cancer survivors with CRCI.

Symptom assessments are currently easier and less costly to obtain compared to neuroimaging. However, we have shown that symptom-based classification combines patients with *distinct profiles of abnormal brain connectivity* within a single category of impaired cognition. Thus, divergent biological mechanisms are considered the same by symptom assessment. Previous research has shown that symptom based cutoff scores are not reliable for classification of cognitive impairment (Luijendijk et al. 2022). For example, symptom classification can be affected by very small differences in performance and artificial inflation of scores related to repeated testing. Self-report cognitive assessments are influenced by situational factors that may be unrelated to cognition, and neuropsychological assessments represent a single period of time within an unrealistic, highly controlled environment. Further, neuropsychological assessments were designed to diagnose severe pathology (e.g., impairments related to lesions, stroke, traumatic brain injury, etc.) but the cognitive impairments associated with chemotherapy are more subtle (Horowitz et al. 2018).

Misclassification could lead to inadequate medical care, psychological distress, and can impact research outcomes. Neuropsychological tests assess the presence of impairments in broad cognitive domains, but do not measure impairments in subprocesses of those domains or the different biological mechanisms which may underly these subprocesses (Horowitz et al. 2018; Luijendijk et al. 2022; Miranda et al. 2021). For CRCI specifically, different chemotherapy regimens may similarly impair cognition but through different biological mechanisms which could impact the effectiveness of different treatment strategies. Simply classifying a patient as cognitively impaired or not after chemotherapy would miss these biological differences that could impact both prognosis and recovery (Horowitz et al. 2018; Miranda et al. 2021).

Treating patients based on their symptoms does not guarantee that the treatment will work. In psychiatric practice, several treatments must be tried to find the best one resulting in unnecessary side effects and delays in symptom improvement. For example, Drysdale et al. (2017) found that depression biotypes predicted responsiveness to transcranial magnetic stimulation which could inform which patients would be the best candidates for this treatment. Additionally, measuring outcomes in CRCI intervention trials based on symptoms alone may yield inaccurate results given the limited sensitivity and specificity these assessments have for CRCI (Horowitz et al. 2018). Biotypes allow us to identify biologically based classifications that could be used as clinical endpoints for intervention trials and to determine more accurate cut off scores for determining impairment from symptom assessments. However, brain MRI is not standard of care for patients with breast cancer and therefore studies examining alternative biotyping approaches such as EEG or functional near-infrared spectroscopy are warranted.

The current study is limited by the sample size which may have prevented us from determining additional separations between biotypes or cognitive symptom-types. We did not have data on other important factors that might distinguish subgroups such as socioeconomic status, physical activity levels, employment status, use of compensatory strategies, etc. Alternative biotyping and symptom-typing methods may yield different results and therefore these findings need to be replicated in future studies to determine reliability. Finally, this study only includes breast cancer survivors who have completed chemotherapy, and therefore findings may differ for those treated for other cancer types or with other therapeutic strategies without chemotherapy (radiation, surgery, etc.). Despite these limitations, our findings provide further evidence that biologically based syndrome classification may be superior to symptom-based classification in improving diagnosis and treatment planning. Further research is needed regarding biotype expression in CRCI to determine characteristics associated with risk and resilience.

## Data Availability

The datasets generated and analyzed during the current study are not publicly available due the fact that they constitute an excerpt of research in progress but are available from the corresponding author on reasonable request.
